# Hidden Diversity in an Antarctic Algal Forest: Metabolomic Profiling Linked to Patterns of Genetic Diversification in the Antarctic Red Alga *Plocamium* sp.

**DOI:** 10.3390/md19110607

**Published:** 2021-10-27

**Authors:** Andrew J. Shilling, Sabrina Heiser, Charles D. Amsler, James B. McClintock, Bill J. Baker

**Affiliations:** 1 Department of Chemistry, University of South Florida, 4202 E. Fowler Ave., Tampa, FL 33620, USA; ashillin@mail.usf.edu; 2 Department of Biology, University of Alabama at Birmingham, 1300 University Blvd., Birmingham, AL 35233, USA; heiser@uab.edu (S.H.); mcclinto@uab.edu (J.B.M.)

**Keywords:** halogenated monoterpenes, metabolite diversity, haplotype diversity, *Plocamium*, Antarctica

## Abstract

The common Antarctic red alga *Plocamium* sp. is rich in halogenated monoterpenes with known anticancer and antimicrobial properties and extracts of *Plocamium* sp. have strong ecological activity in deterring feeding by sympatric herbivores. *Plocamium* sp. collected near Anvers Island, Antarctica showed a high degree of secondary metabolite diversity between separate individuals. GC/MS results revealed 15 different combinations of metabolites (chemogroups) across individuals, which were apparent at 50% or greater Bray–Curtis similarity and also clearly distinguishable by eye when comparing chromatographic profiles of the secondary metabolomes. Sequencing of the mitochondrial *cox*1 gene revealed six distinct haplotypes, of which the most common two had been previously reported (now referred to as Haplotypes 1 and 2). With the exception of one individual, three of the chemogroups were only produced by individuals in Haplotype 1. All the other 12 chemogroups were produced by individuals in Haplotype 2, with five of these chemogroups also present in one of the four new, less common haplotypes that only differed from Haplotype 2 by one base pair. The functional relevance of this metabolomic and genetic diversity is unknown, but they could have important ecological and evolutionary ramifications, thus potentially providing a foundation for differential selection.

## 1. Introduction

Species of the red algal genus *Plocamium* are globally distributed members of the family Plocamiaceae. *Plocamium* spp. are known to produce an array of halogenated monoterpenes that tend to be more chlorinated in comparison to compounds produced in other taxa of red macroalgae. Several chemical investigations into this genus began around the world in the 1970s due to its high abundance in many cooler-water coastal areas and improvements in SCUBA equipment [1,2]. There are a variety of scaffolds produced across the genus, some of which seem to be unique to certain species, while others are more commonly produced across different species. The most ubiquitously occurring compounds across the genus are simple linear polyhalogenated monoterpenes e.g., [3]. Cyclic monoterpenes are also frequently found and so are oxidized halogenated monoterpenes e.g., [4,5,6].

Perhaps the most common halogenated monoterpenes reported from *Plocamium*, and certainly the most numerous secondary metabolites reported from the genus, are from *P.* “*cartilagineum*” [3]. While this taxon is recorded at many places in the world, it is known to be polyphyletic, encompassing several true species [7,8,9], which may explain the relatively large number of distinct compounds reported from it. Examples of *P.* “*cartilagineum*” compounds include a wide variety of both cyclic and acyclic halogenated metabolites [3] as well as unusual oxidized metabolites such as monoterpene aldehydes and hydroxy-bearing pyranoids and furanoids [10,11,12].

Algae previously identified as *P.* “*cartilagineum*” have a circumpolar distribution in Antarctica [13,14] and are often one of the most abundant red algae e.g., [15,16,17,18,19] in the lush macroalgal forests that occur along the northern portion of the western Antarctic Peninsula (WAP) [13,20]. Historically, several *Plocamium* spp. were reported from Antarctica but, more recently, it was hypothesized that these were all just morphological variations of the same entity [21], which was since corroborated by molecular taxonomic data [22]. Although we and others have previously used the name *P.* “*cartilagineum*” for *Plocamium* collected along the WAP, it has been known for some years that the WAP entity is genetically distinct from non-Antarctic *Plocamium* spp. [21,22,23,24], and hereafter, we refer to the entity as *Plocamium* sp. Despite the remoteness of the WAP, *Plocamium* sp. is well studied and is chemically rich, with numerous linear, cyclic, and pyranoid monoterpenes exhibiting various patterns of halogenation [11,25,26,27,28,29]. A number of these compounds have ecological relevance as feeding deterrents toward sympatric macroalgal consumers, and many have promising antibiotic and insecticidal activity [25,28,30,31,32,33]. We recently reported that nine halogenated monoterpenes from WAP *Plocamium* sp. collected near Palmer Station (United States Antarctic Program) on Anvers Island are cytotoxic to cervical cancer cells [29] and that one also inhibits the growth of vancomycin-resistant *Enterococci faecium* [25].

Elsewhere, significant chemical differences between and within populations of *P.* “*cartilagineum*” have been reported for California [34,35], Chile [36,37], and New Zealand [38]. A previous study on the WAP showed that the chemical diversity of this alga is high, resulting in chemical phenotype diversity, where specific chemotypes produce different halogenated terpenes with variable relative abundances, which are hereafter referred to as chemogroups [23]. Patterns of chemogroup variability have been linked through metabolomics with site-specificity, presumably driven by ecological interactions [3,23,30]. In 2013, Young et al. found two haplotypes and five chemogroups among 21 individual algal specimens collected in triplicate from seven different sample sites around Palmer Station [23]. Over 50 peaks were apparent on chromatograms of pooled individuals, most of which displayed halogen isotopic distribution patterns that were indicative of the polyhalogenated monoterpenes that *Plocamium* spp. are known to produce [23]. The two groups, while genetically distinct, were not different enough to be considered separate species under current taxonomic practices and are, therefore, best described simply as different haplotypes [23]. Correlations between haplotypic diversity and secondary metabolite diversity were hypothesized (but not tested) in another red algal genus, *Portiera* [39], which is known to have differences in secondary metabolites between the separate life history stages [40]. However, to our knowledge, *Plocamium* sp. has the only confirmed report of variation in secondary metabolites between haplotypes in red macroalgae. In five out of the seven sites sampled by Young et al., all three individual *Plocamium* sp. were members of the same chemogroup, and in the other two cases, the one individual that differed from the other two was also a member of the other haplotype [23].

As noted, from only 21 individual *Plocamium* sp., Young et al. [23] identified over 50 apparent compounds forming five different chemogroups, which were divided into two different haplotypes. We hypothesized that more extensive collections of *Plocamium* sp. in the Palmer Station area would identify additional chemical and genetic diversity in this ecologically important and secondary metabolite-rich species. Herein, we characterize a total of 15 distinct chemogroups with qualitatively and/or quantitatively distinct combinations of linear and cyclic halogenated monoterpenes, as well as four additional, albeit less common, haplotypes beyond the two previously reported.

## 2. Results and Discussion

**Figure 1:**
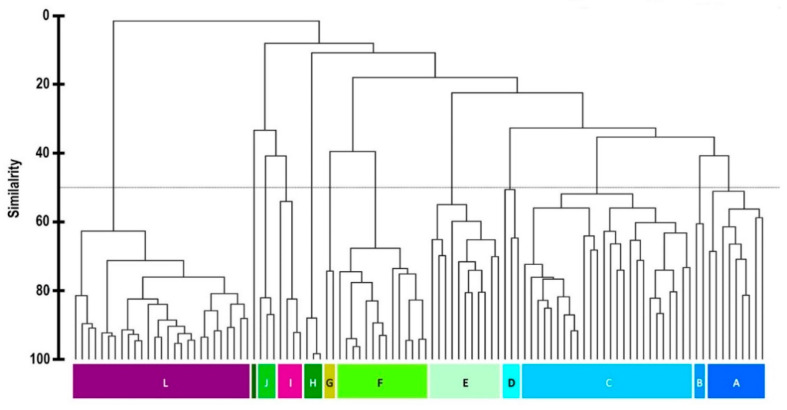
Bray–Curtis similarity dendrogram showing the relationship of the metabolomic profiles between *Plocamium* sp. individuals collected in the 2016 field season. Each vertical line at the bottom represents one individual alga. Algae clusters with greater than 50% similarity (dotted line) were assigned as chemogroups A through L.

**Figure 2:**
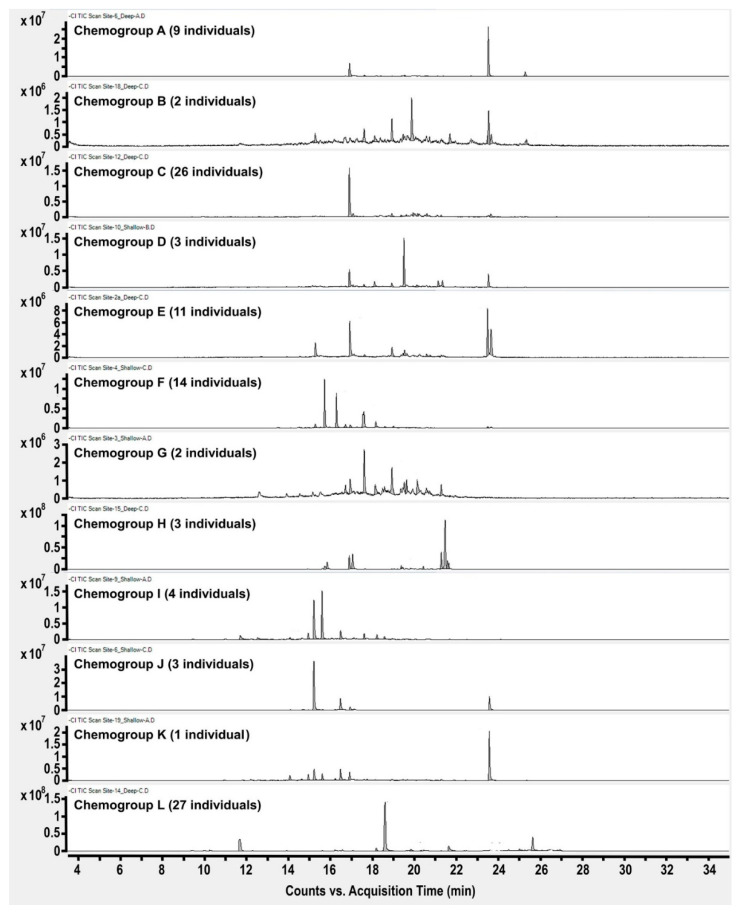
GC/MS (NCI) chromatographic profiles of secondary metabolome of twelve chemogroups of *Plocamium* sp. from the 2016 collection.

In order to gain a greater understanding of the chemical variability, a metabolomic investigation was initiated with a new and larger field collection from within an approximately 3.5 km radius of Palmer Station, Antarctica, in 2016. Individual *Plocamium* sp. were collected in triplicate from 19 different sites (Figure S1) at two separate depths (where possible) in an attempt to find new chemogroups and assess whether depth could also be a factor in determining the chemogroup of an individual at a given site. Well over 60 compounds were apparent in GC/MS results from this much larger collection. Bray–Curtis similarity analysis was performed on the GC/MS results using 67 compounds, identified between 9 and 28 min, that exceeded a threshold cutoff of approximately 1%. This analysis indeed revealed several new chemogroups, for a total of 12 that were apparent at 50% or greater similarity (Figure 1). Each chemogroup contained a unique base peak, representing a distinct major compound, as one of the defining characteristics of each group (Figure 2). The 12 chemogroups identified in 2016 were assigned with a lettering system from A to L based on the sort order of Figure 1. Four of the five chemogroups described by Young et al. [23] from 2012 collections in the same area were also present in these 2016 collections (Young et al. chemogroup 1 is the present L, 2 is C, 4 is D, and 5 is I).

The results of this analysis are similar to the previous findings, but indicate that the relationship between collection site and chemogroup was more variable than described in the original 2013 report [23]. This is not surprising considering the much larger sample size. Fifteen out of the 35 triplicate specimens (43%) showed site- and depth-specificity by only having one chemogroup present, albeit the specific chemogroup varied. In comparison, five out of seven previously studied triplicate specimens (73%) showed site-specificity (depth was not studied). However, 33 out of the 35 triplicate specimens (94%) had no more than two different chemogroups present, compared to seven out of seven (100%) in the smaller study. While a larger sample size could account for some of the increased variability between the studies, depth, as the new variable examined within this experimental design, seemed to show a strong relationship with distribution. Only one out of the 15 study sites (7%), at which both shallow and deep collections were made, had only a single chemogroup at both depths. Just four out of those 15 sites showed at least two individuals from both depths (four in total between depths) in the same chemogroup (27%). This suggests that differences between depths at sites are common, and that depth could be a more important factor than collection site in driving chemical diversification. A number of important abiotic and biotic factors co-vary with depth, particularly solar irradiance and water motion, but also sometimes other factors such as nutrient concentrations or predation pressure. The relevance of these other factors to apparent depth patterns in chemogroups requires further analysis.

**Figure 3:**
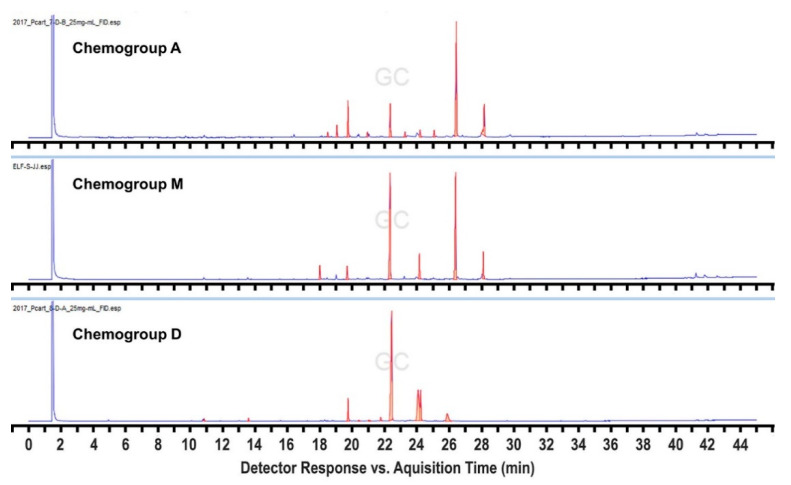
FID chromatographic profiles comparing the secondary metabolomes of chemogroups A and D to the “hybrid” chemogroup M, which shares the characteristic peaks of each in relatively equal abundance.

**Figure 4:**
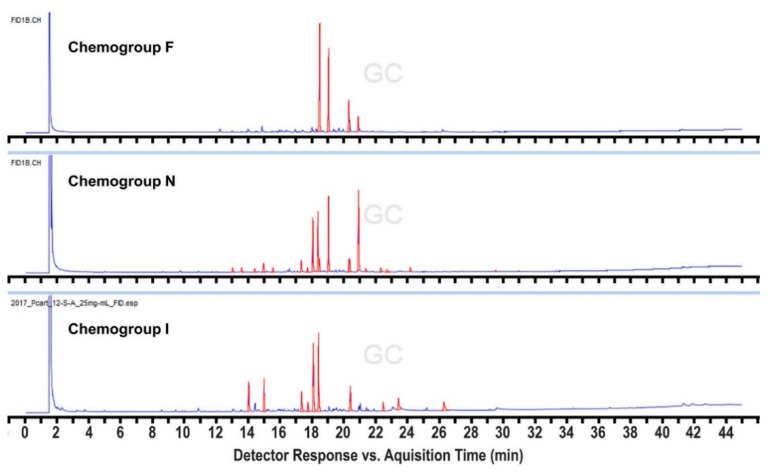
FID chromatographic profiles comparing the chemically populated “hybrid” chemogroup N, which displays relatively high abundances of the characteristic peaks of chemogroups F and I, in addition to its own unique base peak.

**Figure 5:**
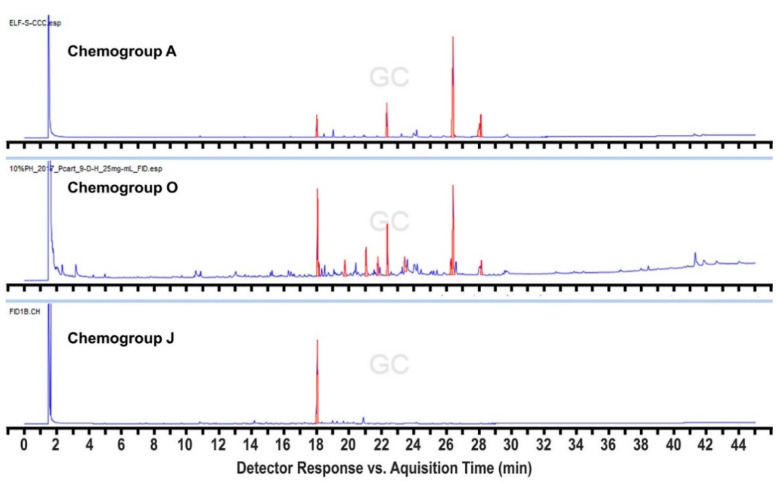
FID chromatographic profiles comparing the secondary metabolomes of chemogroups A and J to the “hybrid” chemogroup O, which shares the characteristic peaks of each in relatively equal abundance.

In order to expand this survey, additional collections were made in 2017 and 2018 from a subset of the 2016 sites, but also from four additional sites within 3.5 km of Palmer Station (Figure S1), as well as six others up to 17 km away. We also sampled more methodically and intensively across a broader depth range at several of the sites. This resulted in the identification of three additional “hybrid” chemogroups designated as M, N, and O. Most importantly, a commonly found hybrid chemogroup, designated as M, was identified, which shares compounds with both chemogroups A and D from the original analysis, but tends to cluster with chemogroup D when subjected to Bray–Curtis similarity analysis. Individuals within this hybrid chemogroup typically produce both characteristic metabolites identified in chemogroups A and D, respectively, in equal abundance and have, therefore, been distinguished as constituting their own chemogroup (Figure 3). Chemogroup M could potentially represent individuals that are transitioning from one chemical phenotype to the other. This could also be the case for chemogroup N, which shares compounds with both chemogroups F and I, and chemogroup O, which shares compounds with both chemogroups A and J, although these hybrids were found far less frequently (Figure 4 and Figure 5). The intensive collections at these 2017 and 2018 sites revealed a great deal of variation in patterns of depth distribution within and between sites, which will be reported separately.

Across the three field seasons, a total of 1397 individuals were analyzed for assignments to the 15 chemogroups. A total of nine known halogenated monoterpenes [3,11,25,26,27,28,29] were identified in the 15 chemogroups (Table 1, Figures S2–S5) in addition to over 60 unidentified compounds with unique retention times (Figures S2–S5). Previously reported compounds were identified within extracts by matching retention times and NCI fragmentation patterns to isolated standards on hand from previous GC/MS- and NMR-guided fractionation efforts of *Plocamium* sp. bulk collections [29]. Two of the unknown compounds are presumed to be epi-plocamene D and plocamiopyranoid A, which were previously described from Palmer Station area *Plocamium* sp. [25], but were identified here solely based on their GC/MS profile. Based on retention times in the GC/MS chromatograms, a fragmentation pattern that was consistent with that expected of plocamiopyranoid A was present as a trace compound in chemogroup L. NCI profiles that were consistent with that expected of epi-plocamene D were present as minor or trace components of chemogroups B, C, D, E, and G. None of these profiles were characteristic of any of the chemogroups, and we cannot rule out that they were from isomers of the two previously identified compounds.

**Figure 6:**
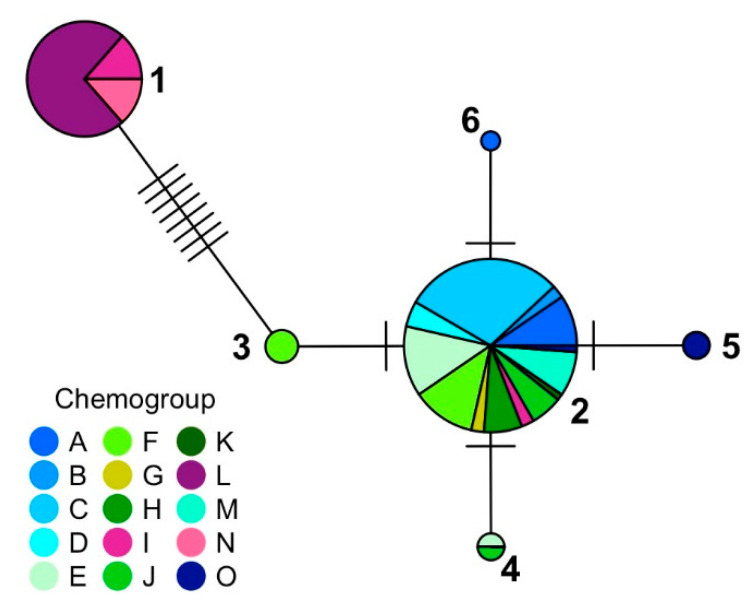
Median joining network based on samples from this study and [23] using the mitochondrial *cox*1 gene in *Plocamium* sp. Sizes of the network nodes represent the number of individuals of each haplotype. Different colors represent the different chemogroups and the purple vs. green-to-blue shades represent the chemogroups produced by the two main haplotypes, Haplotypes 1 and 2, respectively.

The two most common haplotypes (Figure 6) were Haplotypes 1 (37 individuals) and 2 (84 individuals), which were previously described by Young et al. [23] as Haplotypes A and B, respectively. Four new haplotypes were also identified: Haplotype 3, with three individuals (all chemogroup F); Haplotype 4, with two individuals (chemogroup E and J); Haplotype 5, with two individuals (both chemogroup O); and Haplotype 6, with one individual (chemogroup A). With one exception, chemogroups I, L, and N were only produced by individuals in Haplotype 1. All the other chemogroups were produced by individuals in Haplotype 2 through 6, and no chemogroup was unique to Haplotypes 3 through 6. Haplotype 2 was most common around Palmer Station. Guillemin et al. [24] sequenced *Plocamium* sp. from five different sites along the Antarctic Peninsula and their dominant haplotype was only one base pair different from Haplotype 2, the most common in this study.

In summary, we documented a remarkable degree of secondary metabolite diversity between individuals of a single species from a relatively small geographic area. Populations of *Plocamium* spp. from other parts of the world are known to produce multiple halogenated monoterpenes and related compounds [10,11,12,34,35,36,37,38], but individuals from these populations have not been analyzed separately as we have undertaken in the present study. Consequently, it is not possible to know if the high diversity we have documented at the level of individuals is unique to *Plocamium* sp. from the WAP or is more widespread but unrecognized because chemical analyses in other areas have been performed with pooled individuals of differing individual chemogroups.

Regardless, an important question remains as to what biotic and/or abiotic factors have selected for this intraspecific chemical diversity? WAP *Plocamium* sp. is chemically defended from a variety of herbivores [14,30,33,41,42] but is preyed upon by at least one sympatric herbivore [14,42]. In closely related species of terrestrial plants, it is thought that neighboring plants with differing chemical defensive compounds are less vulnerable to common herbivores, and thus, that possessing rare defense phenotypes increases fitness [43,44,45]. Perhaps a similar benefit could be occurring within *Plocamium* sp. if nearby individuals are elaborating differing chemogroups that are more or less effective against different potential herbivores. Testing this hypothesis would require additional information on the fine-scale distribution of the chemogroups as well as on the relative effectiveness of the different chemogroups in deterring potential herbivores.

The distribution of the 15 chemogroups into two distinct haplotypic groups (1 vs. 2–6) indicates that there is at least some genetic driver underpinning the chemical diversity. The *cox*1 marker is commonly used to distinguish both inter- and intraspecific differences in macroalgae, but more polymorphic, nuclear markers, such as microsatellites or single nucleotide polymorphisms (SNPs), are necessary to reveal correlations between genetic and chemical diversity. Consequently, we cannot discount the possibility that there is a genetic basis, reflected by their differing chemogroups, for many or most of the phenotypic differences between individuals. Testing that hypothesis, however, would require more extensive genetic analyses than is possible with any single gene marker. The great diversity of secondary metabolites produced by prey has long been thought to be the result of escalating coevolution—sometimes called an “evolutionary arms race”—between prey and predators [46,47], and Ehrlich and Raven [46] postulated that increasing chemodiversity and biodiversity are directly related. They hypothesized that the coevolution of prey defenses and predator counter-defenses can allow both groups to colonize new ecological niches where they can diversify in the relative absence of other predators or of competitors (the “escape and radiate” hypothesis; cf. [48,49]). As discussed above, there is a great deal of cryptic speciation world-wide within taxa currently or previously described as *P.* “*cartilagineum*”, including the WAP *Plocamium* sp., and there is also a great deal of chemical diversity in the algae from different locations. Assuming that different chemogroups have differing ecological benefits and/or differing physiological costs, and knowing that they have a genetic basis at least in part, it is possible that the rich chemodiversity has provided a template for differential selection that underlies the observed worldwide cryptic speciation in *Plocamium*. If so, the genetic and phenotypic variation in the WAP *Plocamium* sp. may well represent an intermediate stage in such cryptic speciation.

## 3. Materials and Methods

### 3.1. Biological Material

*Plocamium* sp. individuals were collected from 19 sites in February through April 2016, and from additional sites in February through May 2017 and February through June 2018. The collections were cleaned of foreign materials when present and frozen at −20 °C with subsequent transport to the University of South Florida for analysis. Individuals were collected from a total of 23 sites within a 3.5 km radius of Palmer Station, Antarctica (64°46.5′ S; 64°03.3′ W; Figure S1), and an additional six sites within approximately 17 km of Palmer Station in the Casey Islands, Joubin Islands, and Wauwerman Islands. In 2016, at most sites, three individuals were collected from each of two depths. In 2017 and 2018, up to 15 individuals each were collected from up to nine separate depths per site.

### 3.2. General Procedures

Solvents were obtained from Fisher Scientific Co. (Pittsburgh, PA, USA) and were of HPLC grade (>99% purity) unless otherwise stated. GC/HRMS analysis was performed on an Agilent (Santa Clara, CA, USA) 7890A GC coupled to an Agilent 7200 accurate mass QToF with negative chemical ionization utilizing methane as the reagent gas on a Zebron ZB-5HT Inferno (30 m × 0.25 mm, 0.25 μm film thickness) column. Metabolite identity was determined by retention time and mass spectrum characteristics (m/z and isotopic distribution pattern) as previously determined [23,29].

### 3.3. Metabolomic Analysis of Plocamium sp.

Individual *Plocamium* sp. collected in triplicate from each of 19 sites, at 2 separate depths, during our 2016 field season were subjected to 2 days of extraction (wet) in 3:1 dichloromethane/methanol. The organic layer of each algal extract was filtered through a 0.45 μm PTFE membrane and concentrated under a stream of N_2_ gas. Dried extracts were each prepared at 1 mg/mL in MeOH for GC/MS analysis using an Agilent 7980A GC interfaced to an Agilent 7000 series QqQ mass spectrometer operating in chemical ionization (CI) mode, using methane at 2 mL/min as the ionization gas. Injections of 1 μL of the algal extract solution were vaporized on the preheated splitless inlet at 250 °C, then introduced onto an HP-5ms column (30 m × 0.25 mm i.d.) using a 35 min temperature gradient (initial oven temperature of 100 °C, held for 2 min, heated to a final temperature of 250 °C at a rate of 5 °C/min, then held at final temperature for a further 3 min). Helium was used as a carrier gas at a constant flow rate of 1 mL/min [23].

Metabolomic analysis of *Plocamium* sp. individuals collected during the 2017 and 2018 field seasons was performed on either an Agilent 6980A or 7980A GC, equipped with a flame ionization detector using a Zebron ZB-5HT Inferno column (30 m length × 0.25 mm ID × 0.25 mm film thickness). Helium was used as the carrier gas, while hydrogen and compressed air were used as the combustion gasses in the FID, with nitrogen used as the sweeping gas. Extractions were accomplished using the same methodology listed above and dried extracts were each prepared at 25 mg/mL in MeOH for GC analysis. Injections of 1 μL of the algal extract solution were vaporized on the preheated multimode inlet (MMI) inlet at 250 °C, then introduced onto the column using a 45 min temperature gradient (initial oven temperature of 100 °C, held for 2 min, heated to a final temperature of 300 °C at a rate of 5 °C/min, then held at final temperature for a further 3 min). The carrier gas was kept at a constant flow rate of 1.2 mL/min, with hydrogen supplied to the FID at 40 mL/min and mixed with compressed air supplied at 400 mL/min and a sweeping gas (N_2_) flow rate of 25 mL/min.

### 3.4. Statistical Analysis of Plocamium sp. Metabolomics

The GC/MS data were analyzed in Agilent’s MassHunter Qualitative B6.01 (Agilent, Santa Clara, CA, USA), where a total of 67 compounds were identified between 9 and 28 min throughout the 105 different samples, with all nine elucidated structures represented (identified with standards) as well as two other previously reported compounds likely present. The integrated peak areas of the 10 most prominent peaks within each site were normalized to yield the relative abundance (%) of each compound. These relative abundance (%) values were analyzed for resemblance using Bray–Curtis similarity and clustered using the group average with PRIMER-6 software (PRIMER-E, Auckland, New Zealand). Clusters exhibiting greater than 50% Bray–Curtis similarity were grouped together into 12 different chemogroups [23].

The GC/FID data generated from the 2017 and 2018 field seasons were analyzed in ACD/Labs Spectrus Processor 2016.2 (ACD/Labs, Toronto, Ontario, Canada), where a total of over 75 compounds were identified between 9 and 30 min throughout more than 1200 different samples, and were grouped using both the statistical methodology listed above and qualitatively when needed. Elucidated compounds were identified with standards.

### 3.5. Determination of Haplotypes

Total genomic DNA was extracted from approximately 10–15 mg of dried tissue using one of two kits. For the Qiagen DNeasy^®^ Plant Mini kit, we followed the manufacturer’s recommendations, except for the elution step, in which we eluted in 50 µL autoclaved Milli-Q water rather than the elution buffer. For the Machery-Nagel Nucleospin^®^ Plant II kit, we used PL1 for the lysis step at room temperature and eluted in 100 µL of autoclaved Milli-Q water. A total of 129 thalli were amplified at the mitochondrial *cox*1 gene using the primers GazF1 (5′TCAACAAATCATAAAGATATTGG3′) [50] and GWSRx (5′ACTTCTGGRTGICCRAARAAYCA3′) [51]. We used variable DNA dilutions between 1:50 and 1:500, where most samples were amplified at 1:100. Following [1], we used the following PCR conditions, for a total volume of 25 µL:2.5 µL of DNA, 500 nM of each primer, 1× Promega 5× colorless GoTaq^®^ Flexi buffer, 2.5 mM of MgCl_2_, 250 µM of dNTPs, and 0.5 units of Promega GoTaq^®^ Flexi DNA Polymerase. The PCR program was as follows: 94 °C for 2 min, followed by 5 cycles of 95 °C for 30 s, 45 °C for 30 s, and 72 °C for 1 min, followed by another 35 cycles of 95 °C for 30 s, 46.5 °C for 30 s, and 72 °C for 1 min, with a final extension at 72 °C for 5 min [23].

If amplification for more than one DNA concentration was successful, the product was pooled and purified using one of two methods. Using the Promega Wizard^®^ SV gel clean-up kit according to the manufacturer’s protocol, we obtained 5 µL of purified PCR product and added 1 µL of 5 µM forward primer, and this was subsequently sent to the UAB Heflin Center for Genomic Sciences (Birmingham, AL, USA) for Sanger sequencing. For the other method, we added 1 µL ExoSAP-It (Affymetrix, Santa Clara, CA, USA) to 7 µL of PCR product, followed by a 15 min incubation at 37 °C and for 15 min at 80 °C. We mixed 4 µL of 2 µM forward primer and submitted samples to Eurofins Genomics (Louisville, KY, USA) for commercial Sanger sequencing. PCR products with reverse primers were submitted where necessary to confirm base pair calls.

The software 4Peaks (Nucleobytes, Aalsmeer, Netherlands) was used for initial editing of sequences, which were than aligned with the original haplotypes A and B [23] using the software Geneious Prime 2021.2.2 (Biomatters, San Diego, CA, USA). A median joining network [52] was created in R [53] with the adegenet ver. 2.1.3 [54], ape ver. 5.4-1 [55] and pegas ver. 0.14 packages [56]. New haplotypes (found subsequently to those in [23]) were submitted to GenBank with the following accession numbers: OK631662–OK631665.

## 4. Conclusions

As discussed, we documented a high degree of secondary metabolite diversity between individuals of *Plocamium* sp. from a relatively small geographic area. None of our collection sites (Figure S1) yielded all or even most of the 15 identified chemogroups. Had we collected only at one, or even a few, of these sites, we would have missed chemical diversity in the Palmer Station area *Plocamium* sp. Consequently, from a drug discovery perspective, we could have missed possible candidates for further study. This illustrates the value of making collections from as many sites as possible, even if without great spatial separation, to maximize the possibility of discovering important new compounds. Our results, which document that the chemogroups almost exclusively partition between two distinct groupings of the six identified haplotypes, illustrate that genetic differences probably underlie some of the chemical diversity. However, environmental factors, particularly within haplotypes, may also be important.

## Figures and Tables

**Table 1:** Presence of known compounds within chemogroups A–O.

ID	Structure	Characteristic (Major) Metabolite of Chemogroups	Minor Metabolite in Chemogroups (>5% AUC)	Trace Metabolite in Chemogroups (<5% AUC)
1	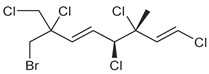	A, M *, O *	D, L	none
2	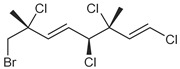	D, M *	A, G, O	E, N
3	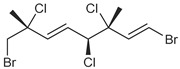	none	D, M	A, N, O
oregonene A	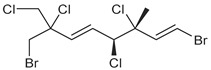	none	A, M	D
anverene A	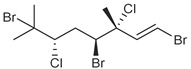	H	none	none
anverene B	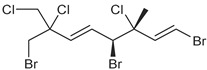	none	none	A, D, M
anverene C	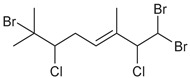	none	B, C, E	F
anverene D	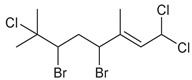	none	none	B, E, G
anverene E	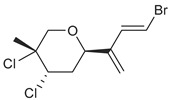	L	none	none

* Compound present in equal abundance with another major metabolite.

## Data Availability

Metabolomics data are contained within the article or Supplementary Material. These data are available in Figure 1, Figure 2, Figure 3, Figure 4 and Figure 5 and Supplementary Figures S2–S5. Gene sequence data are available in a publicly accessible repository that does not issue DOIs. These data can be found at https://www.ncbi.nlm.nih.gov/nuccore/ (accessed on 12 April 2021) under accession numbers OK631662 through OK631665.

## Supplementary Materials

The following are available online at https://www.mdpi.com/article/10.3390/md19110607/s1, Figure S1: *Plocamium* sp. collection sites near Palmer Station. Figure S2: GC/MS (NCI) identification of major components of chemogroup A. Figure S3: GC/MS (NCI) identification of major components of chemogroup D. Figure S4: GC/MS (NCI) identification of major components of chemogroup H. Figure S5: GC/MS (NCI) identification of major components of chemogroup L.
