# Supplementary material for: Hidden Diversity in an Antarctic Algal Forest: Metabolomic Profiling Linked to Patterns of Genetic Diversification in the Antarctic Red Alga Plocamium sp
Source: Mar Drugs. 2021 Oct 27;19(11):607. doi: 10.3390/md19110607 (PMC8622728; doi:10.3390/md19110607)
Supplement: Supplementary file 1 [file marinedrugs-19-00607-s001.zip › marinedrugs-1413543-supplementary.pdf]

Supplementary Information

Hidden Diversity in an Antarctic Algal Forest: Metabolomic Profiling Linked to Patterns of Genetic Diversification in the Antarctic Red Alga *Plocamium* sp.

Andrew J. Shilling[1], Sabrina Heiser[2], Charles D. Amsler[2*], James B. McClintock[2], and Bill J. Baker[1*]

1 Department of Chemistry, University of South Florida, 4202 E. Fowler Ave., Tampa, FL 33620, USA; andrewjshilling@yahoo.com (A.J.S.)

2 Department of Biology, University of Alabama at Birmingham, 1300 University Blvd., Birmingham, AL 35233-1405, USA; heiser@uab.edu (S.H.); mcclinto@uab.edu (J.B.M.)

* Correspondence: amsler@uab.edu (C.D.A.); bjbaker@usf.edu (B.J.B)

Figure S1. Plocamium sp. collection sites near Palmer Station…………………………….. 2
Figure S2. GC/MS (NCI) identification of major components of chemogroup A…………... 3
Figure S3. GC/MS (NCI) identification of major components of chemogroup D…………... 3
Figure S4. GC/MS (NCI) identification of major components of chemogroup H…………... 4
Figure S5. GC/MS (NCI) identification of major components of chemogroup L…………... 4

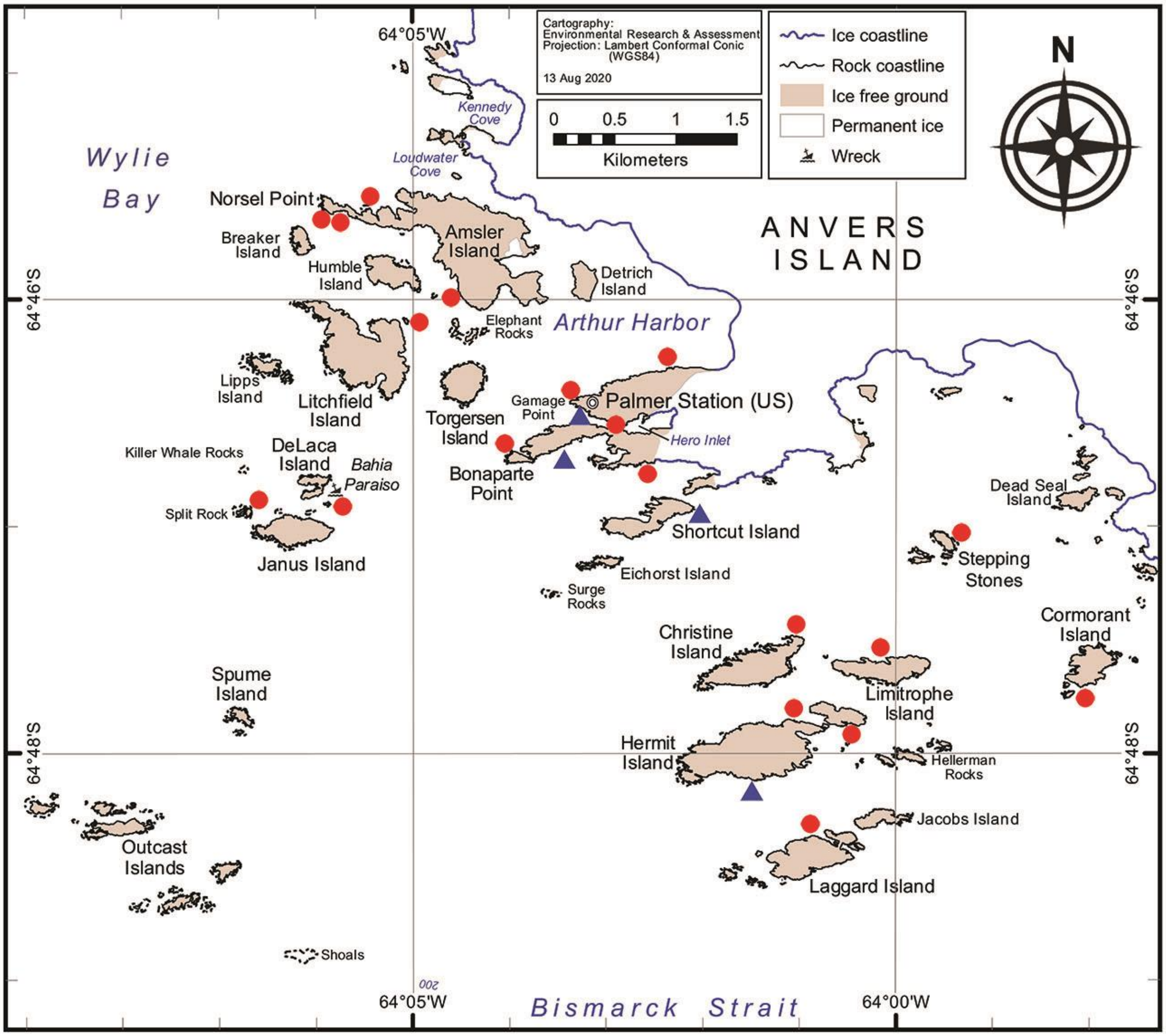

Figure S1. *Plocamium* sp. collection sites near Palmer Station, Antarctica. Red circles indicate collection sites from 2016, most of which were resampled in 2017 and/or 2018. Blue triangles indicate additional collection sites from 2017 and 2018.

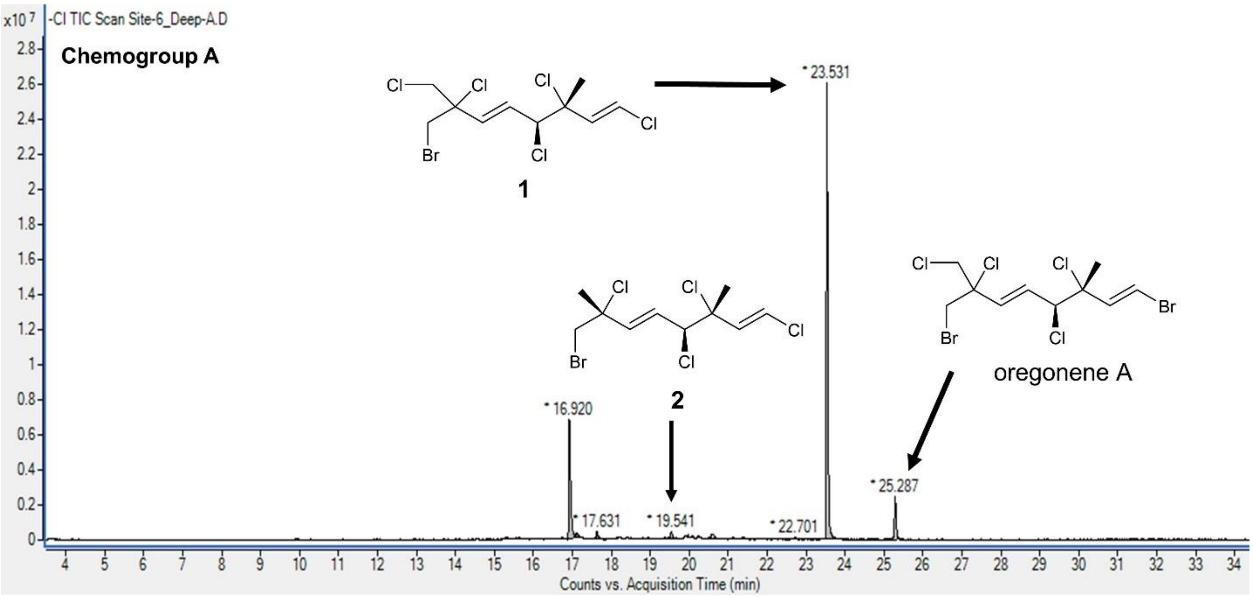

Figure S2. GC/MS (NCI) identification of major components of chemogroup A.

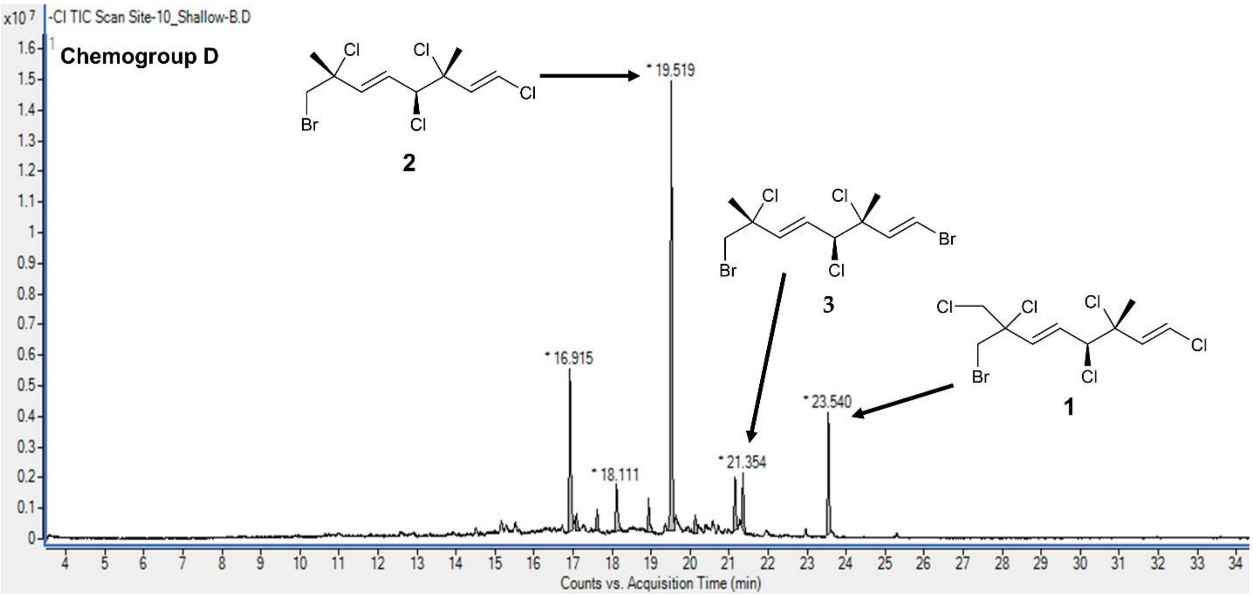

Figure S3. GC/MS (NCI) identification of major components of chemogroup D.

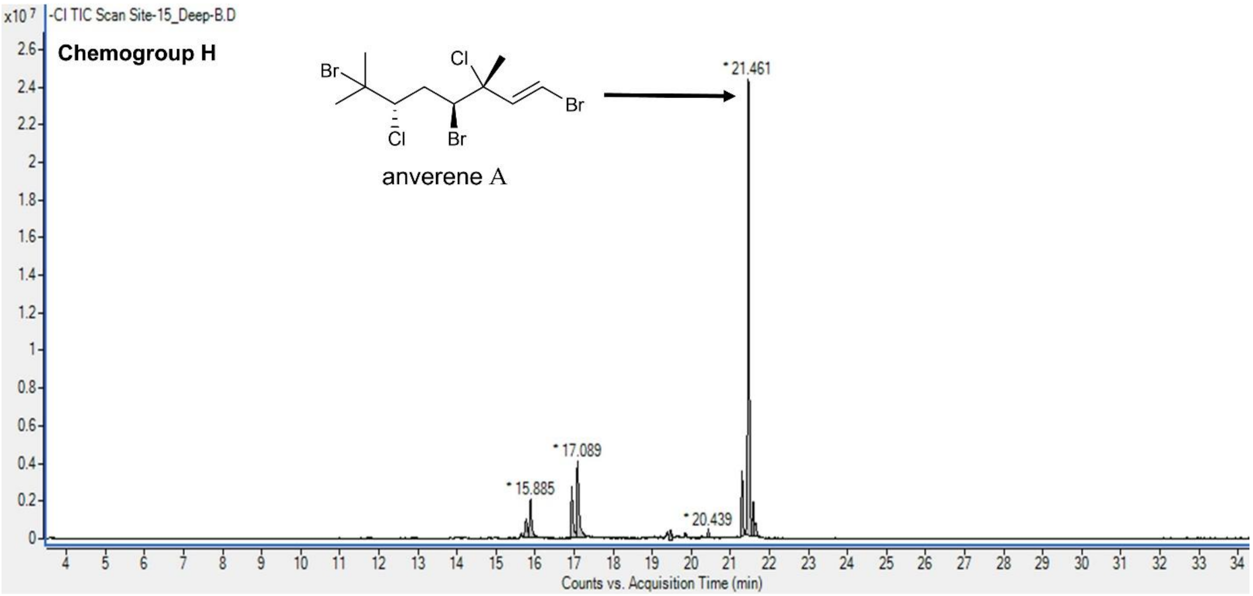

Figure S4. GC/MS (NCI) identification of major components of chemogroup H.

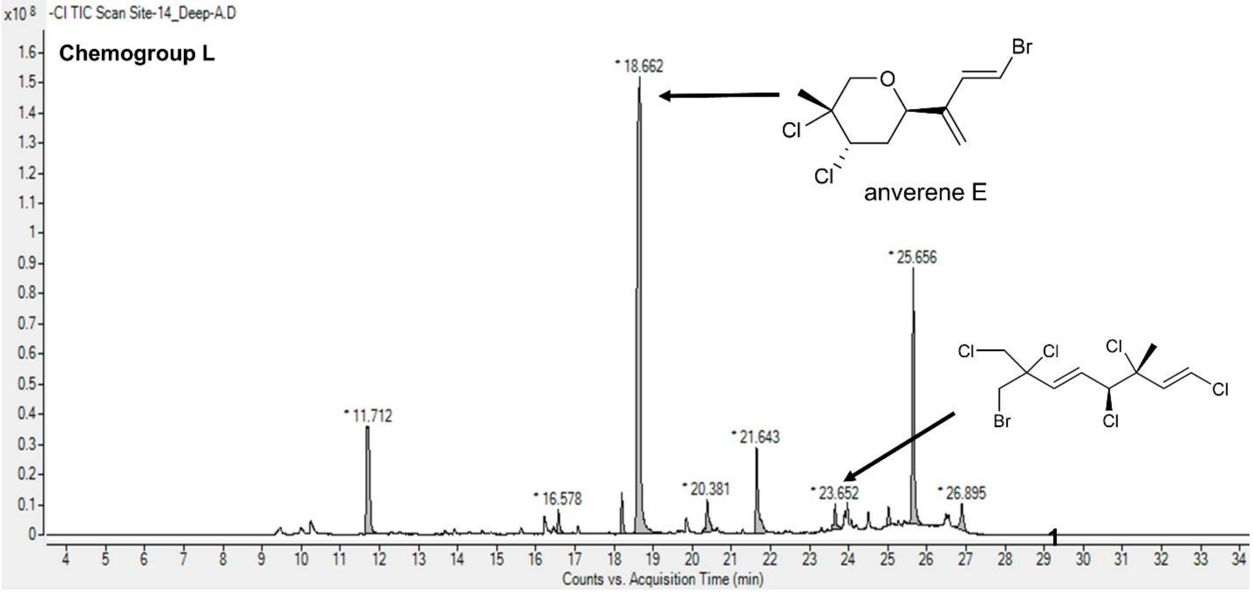

Figure S5. GC/MS (NCI) identification of major components of chemogroup L.
